# Effect of Glyceraldehyde Cross-Linking on a Rabbit Bullous Keratopathy Model

**DOI:** 10.1155/2015/171690

**Published:** 2015-10-05

**Authors:** Mengmeng Wang

**Affiliations:** Hebei Provincial Eye Hospital, Hebei Provincial Ophthalmology Key Lab, Hebei Provincial Institute of Ophthalmology, Xingtai, Hebei 054001, China

## Abstract

*Background*. To evaluate the effects of corneal glyceraldehyde CXL on the rabbit bullous keratopathy models established by descemetorhexis. *Methods*. Fifteen rabbits were randomly divided into five groups. Group A (*n* = 3) is the control group. The right eyes of animals in Groups B,C, D, and E (*n* = 3, resp.) were suffered with descemetorhexis procedures. From the 8th day to the 14th day postoperatively, the right eyes in Groups C and D were instilled with hyperosmolar drops and glyceraldehyde drops, respectively; the right eyes in Group E were instilled with both hyperosmolar drops and glyceraldehyde drops. Central corneal thickness (CCT), corneal transparency score, and histopathological analysis were applied on the eyes in each group. *Results*. Compared with Group A, statistically significant increase in CCT and corneal transparency score was found in Groups B, C, D, and E at 7 d postoperatively (*P* < 0.05) and in Groups C, D, and E at 14 d postoperatively (*P* < 0.05). *Conclusion*. Chemical CXL technique using glyceraldehyde improved the CCT and corneal transparency of the rabbit bullous keratopathy models. Topical instillation with glyceraldehyde and hyperosmolar solutions seems to be a good choice for the bullous keratopathy treatment.

## 1. Introduction

Bullous keratopathy is a condition of overhydration (edema) of the cornea, resulting from endothelial failure [1]. It is characterized by both stromal and epithelial edema; the increase of corneal thickness signifies the aggravation of hydration. The most common reasons associated with this disease include detachment of Descemet's membrane [2], Fuchs endothelial dystrophy [3], and postoperative bullous keratopathy [4]. Clinically, many therapeutic methods have been used to treat bullous keratopathy.

Collagen cross-linking (CXL), introduced by Wollensak et al. [5, 6], is an effective approach to increase the biomechanical strength of the corneal and scleral tissue [7]. By means of a highly localized photopolymerization, corneal CXL can create additional chemical bonds inside the corneal stroma, compact the anterior corneal stroma, and decrease the central corneal thickness [8]. Cross-linking might become a useful tool in the temporary treatment of bullous keratopathy [9]. Glyceraldehyde (C3H6O3) is a simple aldotriose sugar. As a chemical cross-linking agent, glyceraldehyde not only provided excellent efficacy of increasing scleral rigidity by up to 419% [7] but also showed low toxicity on in vitro corneal epithelial and endothelial cell lines [10].

The purpose of the present study was to evaluate the effects of corneal glyceraldehyde CXL on the rabbit bullous keratopathy models, which were established by descemetorhexis.

## 2. Methods

### 2.1. Animals

Fifteen New Zealand adult albino rabbits weighing 2.0–3.0 kg were obtained from the Laboratory Animal Center of Peking University. Before recruiting into the experiment, all rabbits were given a complete ophthalmological and systemic examination to exclude any ocular and body disease. All procedures in the present study were approved by the Ethics Committee of Peking University and were in accordance with the Association for Research in Vision and Ophthalmology (ARVO) Statement for the Use of Animals in Ophthalmic and Vision Research.

### 2.2. Grouping and Treatment Protocols

According to the treatment protocols shown in Table 1, these animals were randomly divided into five groups. Group A is the sham operated control group (only corneal incision, without descemetorhexis, *n* = 3). To establish the bullous keratopathy, the right eyes of animals in the other four groups were suffered with detachment of Descemet's membrane using a descemetorhexis technique [11]. In Group B (*n* = 3), no treatment was applied for the bullous keratopathy postoperatively. In Group C (*n* = 3), hyperosmolar drops (5.00% NaCl) were instilled in the eyes 4 times daily from the 8th day to the 14th day postoperatively. In Group D (*n* = 3), glyceraldehyde drops (0.5 M glyceraldehyde (DL-glyceraldehyde, Wako Pure Chemical Industries, Ltd., Osaka, Japan) and 0.02% benzalkonium chloride [BAC, Wako Pure Chemical Industries, Ltd., Osaka, Japan] in 0.90% NaCl) were instilled in the eyes 4 times daily from the 8th day to the 14th day postoperatively. In Group E (*n* = 3), both hyperosmolar drops and glyceraldehyde drops were combined to be instilled in the eyes 4 times daily from the 8th day to the 14th day postoperatively.

### 2.3. Descemetorhexis Procedure

All operations were performed by the same surgeon (M.W.) under sterile conditions. After the general and topical anesthesia, the right eyes of animals in Groups B, C, D, and E had a self-sealing clear corneal incision (2.0 mm in length and 3.0 mm in width) at the 12 o'clock surgical position of peripheral cornea. A hook was used to strip the surrounding edges of Descemet's membrane (DM) inward toward the center and then they were removed from the anterior chamber. Chloramphenicol eye drops were applied 4 times daily for 3 days preoperatively and 7 days postoperatively.

### 2.4. Pre- and Postoperative Examinations

Preoperatively and at the 7th and 15th day postoperatively, both central corneal thickness (CCT) and corneal transparency were measured on the right eyes of all animals to check their corneal conditions. Ultrasound pachymetry was performed for the CCT using Nidek UP-1000 ultrasonic pachymeter (NIDEK CO., LTD., Gamagori, Aichi, Japan). Corneal transparency was measured by slit lamp biomicroscopy and graded according to a previously published scale [12] from 0 to 4 (0 = no edema, totally transparent; 1+ = slight corneal edema, slight loss of transparency; 2+ = moderate edema, iris details seen; 3+ = intense edema, some iris details seen; and 4+ = very opaque, no iris details seen). All examinations were performed on the animals after their general and topical anesthesia by an independent masked examiner.

### 2.5. Histopathological Analysis

All animals were euthanized using an overdose of pentobarbital at the 15th day postoperatively. The right eyes were immediately enucleated for histopathological analysis. The cornea was bisected vertically in the center at the 12 o'clock position. One-half of the cornea was fixed in 4% neutral buffered formalin; 5.0 *μ*m thin paraffin sections were stained with hematoxylin and eosin (H&E). The specimens were evaluated using a light microscope (Leica DM750, Leica Microsystems GmbH, Wetzlar, Germany) at 100- to 400-fold magnification.

### 2.6. Statistical Analysis

Statistical analysis was performed with JMP 9 statistical package (SAS Institute, Inc., Cary, NC, USA) software. Categorical variables were compared using Pearson's chi-square test. When parametric analysis was possible, one-way ANOVA with Tukey's HSD test was used to compare the results among the different groups/time points; when parametric analysis was not possible, the Kruskal-Wallis test with Steel-Dwass test was used instead. Results with *P* < 0.05 were considered statistically significant.

## 3. Results

Table 2 shows the characteristics of 15 included rabbits in CCT and corneal transparency score preoperatively and postoperatively. The preoperative transparency scores of all eyes in four groups were 0, which means they were totally transparent. There was no transparency change in Group A at all time points. At the 7th day after descemetorhexis procedures, the transparency scores in Groups B, C, D, and E were increased from 0 to 3+ in 3 eyes and from 0 to 4+ in 9 eyes. At the end of the study, the transparency scores were ranging between 2+ and 4+ in Group B and between 1+ and 2+ in Group E.

Table 3 shows the mean CCT values of each group at different preoperative and postoperative time points. Statistically significant increase in CCT was found in Groups B, C, D, and E at 7 days postoperatively and in Groups B, C, and D at 15 days postoperatively (*P* < 0.05). Compared with the CCT value in Group B, statistically significant improvements were found in Groups C, D, and E at end of this study (*P* < 0.05). Although the mean value in Group E was thicker than Group A, there was no statistically significant difference between the two groups at the end of this study (*P* = 0.3435).

**Figure 1:**
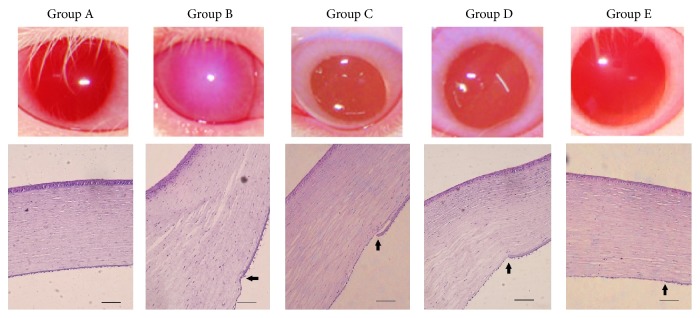
Anterior segment photographs (upper) and corneal photomicrographs (lower, H&E stain; original magnification ×200, bar = 100 *μ*m) of rabbits in each group at 15 days postoperatively. Arrows show the edges of the Descemet membrane in eyes after descemetorhexis procedures.

As was shown in Table 2 and Figure 1, corneal opaque and edema were observed in the corneal stroma 7 days after descemetorhexis procedures, which suggested the bullous keratopathy. Corneal transparency scores were reduced in all eyes of Groups B, C, D, and E at 7 days postoperatively. According to the anterior segment photographs and corneal photomicrographs, corneal transparency and edema condition were observed much better in Groups C and E than in Group B at the end of the study.

## 4. Discussion

Although bullous keratopathy is one of the leading indications, immediate keratoplasty is not a reality in many countries. It has to cost patients a few weeks to years for a suitable corneal tissue from a donor [13]. Thus, several options have been proposed for the bullous keratopathy treatment when patients are waiting for their surgical procedures. Topical hypertonic solutions could yield short-term relief of visual acuity and corneal clarity by reducing the epithelial edema [14]. Recently, a physical CXL technique using ultraviolet (UVA) and riboflavin has been developed to provide temporary improvements in corneal transparency, corneal thickness, and ocular pain [9, 15, 16]. Because of their similar biomechanical efficiency [7], a chemical CXL technique using glyceraldehyde was substituted for the physical CXL technique in the present study as a new attempt for the bullous keratopathy treatment.

In previous studies, transcorneal freezing was performed using a cryoprobe or a brass dowel cooled in liquid nitrogen for establishing the bullous keratopathy models [17, 18]. The cryoprobe or brass dowel should be kept on the corneal surface until the endothelium was affected. After the transcorneal freezing procedures, the severity of endothelial dysfunction could not be accurately reflected by evaluating the postoperative CCT and corneal clarity because of the destruction in overall corneal layers. In the present study, a descemetorhexis technique was performed for establishing the rabbit bullous keratopathy models, which was not intraoperative damage to the epithelium and stroma. It was found that the average values of postoperative CCT were almost 2-3 times thicker than the preoperative levels. The corneal edema and opacity were observed in all rabbit eyes with descemetorhexis procedures at 7 days postoperatively. All these biological and histopathological results proved the efficiency of the descemetorhexis technique in establishing the rabbit bullous keratopathy models.

Both hyperosmolar and glyceraldehyde were proved to be effective in reducing the CCT and corneal transparency scores of rabbit bullous keratopathy models in the present study (*P* < 0.05). Although there was no statistically significant difference among three treatment groups (Groups C, D, and E), the largest improvement in CCT and corneal transparency scores was observed in Group E. It seemed that the hyperosmolar effect of 5.00% NaCl solution and the CXL effect of glyceraldehyde solution were combined in Group E. When the hyperosmolar effect makes the corneal collagen fibers gather together, CXL effect could be much easier to be applied. To verify this combination and improve the topical solution, more bullous keratopathy animals and examination parameters should be included for long-term studies in the future.

The present chemical CXL technique using glyceraldehyde for the bullous keratopathy treatment has the following advantages. First, the toxicity level of glyceraldehyde has preliminarily been proven to be lowest among several chemical CXL agents [10]. Until now, no side effect was reported in previous glyceraldehyde CXL studies involving human [7], porcine [19], guinea pig [20], and rabbit [21, 22] eyes. Second, compared with the invasive physical CXL surgery, no corneal deepithelialization during the glyceraldehyde CXL may yield less postoperative discomfort [23] and complications (such as haze, infective keratitis, and reduction of corneal thickness) [24]. Third, chemical CXL technique is more convenient to be applied. Topical glyceraldehyde solution can be instilled by patients themselves several times daily for a long treatment period.

Nevertheless, the following limitations of the current study should be noted. First, the limited animal sample cannot elaborate information about the long-term efficacy and safety of the current CXL technique. Second, the current rabbit bullous keratopathy model was still different from human cases in clinical settings. Third, because of the lack of corneal glyceraldehyde CXL previously, the only glyceraldehyde concentration (0.5 M) in the present study was chosen according to several scleral CXL studies [19–22]. Finally, although a 24-hour exposure to glyceraldehyde has been proved to be safe for cultured human corneal epithelial cells and bovine corneal endothelial cells [10], long-term safety of this agent was still unknown. Further studies using more animal models and human cases are needed to set up a long-term safe and effective protocol of corneal glyceraldehyde CXL for bullous keratopathy treatment.

In sum, chemical CXL technique using glyceraldehyde improved the CCT and corneal transparency of the rabbit bullous keratopathy models established by descemetorhexis. Topical instillation with glyceraldehyde and hyperosmolar solutions seems to be a good choice for bullous keratopathy patients as a temporary therapeutic measure when they are waiting for the keratoplasty.

## Figures and Tables

**Table 1:** Treatment protocols for the eyes in each group.

Groups	Eyes	Descriptions
A	*n* = 3	Sham operated control
B	*n* = 3	Descemetorhexis
C	*n* = 3	Descemetorhexis + hyperosmolar eye drops
D	*n* = 3	Descemetorhexis + glyceraldehyde eye drops
E	*n* = 3	Descemetorhexis + hyperosmolar eye drops + glyceraldehyde eye drops

**Table 2:** Characteristics of 15 rabbits in central corneal thickness (CCT) and corneal transparency score.

Groups	Animals	CCT, *μ*m			Corneal transparency score		
		Pre	7 d	15 d	Pre	7 d	15 d
A	1	373	375	378	0	0	0
	2	354	350	357	0	0	0
	3	391	385	390	0	0	0

B	4	346	1020	975	0	4	4
	5	368	871	804	0	3	2
	6	359	905	783	0	4	3

C	7	375	879	497	0	3	1
	8	337	1010	652	0	4	2
	9	380	935	585	0	4	2

D	10	352	986	614	0	4	2
	11	347	1019	657	0	4	3
	12	359	873	489	0	3	1

E	13	338	1014	496	0	4	1
	14	343	902	449	0	4	1
	15	389	1040	534	0	4	2

Pre, before descemetorhexis surgery; 7 d, 7 days after descemetorhexis surgery; 15 d, 15 days after descemetorhexis surgery.

**Table 3:** Central corneal thickness (CCT) of each group at different time points pre- and postoperatively.

Groups	CCT			*P* values	*P* values of post hoc comparison		
	Pre	7 d	15 d	Among three time points	Pre versus 7 d	7 d versus 15 d	Pre versus 15 d
A	372.67 ± 18.50	370.00 ± 18.03	375.00 ± 16.70	0.9428	NS	NS	NS
B	357.67 ± 11.06	932.00 ± 78.08^*∗*^	854.00 ± 105.31^*∗*^	0.0002	0.0002	NS	0.0005
C	364.00 ± 23.52	941.33 ± 65.73^*∗*^	618.50 ± 47.38^*∗*,§^	<0.0001	<0.0001	0.0008	0.0115
D	352.67 ± 6.03	959.33 ± 76.57^*∗*^	586.67 ± 87.27^*∗*,§^	<0.0001	<0.0001	0.0012	0.0125
E	356.67 ± 28.11	985.33 ± 73.33^*∗*^	493.00 ± 42.58^§^	<0.0001	<0.0001	<0.0001	0.0407

Pre, before descemetorhexis surgery; 7 d, 7 days after descemetorhexis surgery; 15 d, 15 days after descemetorhexis surgery; NS, no significance. ^*∗*^Statistically significant difference compared with the value in Group A at the same time point; ^§^statistically significant difference compared with the value in Group B at the same time point.
